# Exploring care adoption intention among older adults using XGBoost: a rural case in Anhui, China

**DOI:** 10.3389/fpubh.2026.1920052

**Published:** 2026-08-20

**Authors:** Jingli Liu, Ziwei Yang

**Affiliations:** 1 School of Humanities and Social Sciences, Anhui University of Science and Technology, Huainan, China; 2 School of Management, Hefei University of Technology, Hefei, China

**Keywords:** adoption intention, rural older adults, smart mutual-aid care, technology acceptance model, XGBoost

## Abstract

**Introduction:**

With the development of digital rural construction and rural mutual-aid care for older adults, smart mutual-aid care for older adults has become an important way to improve rural pension services, yet low digital literacy and insufficient adoption intention among rural older adults hinder the popularization of smart care services for older adults.

**Methods:**

Based on the Technology Acceptance Model, this study collected 320 valid questionnaires from rural older adults in Anhui Province and combined traditional statistical approaches with explainable machine learning to explore factors affecting service adoption intention.

**Results:**

The questionnaire possessed good reliability and validity with significant correlations across all core variables; perceived ease of use and perceived usefulness significantly boosted adoption intention, while social support lost statistical significance due to collinearity suppression. Perceived ease of use served as the dominant driving factor, social support contributed positively independently, the explanatory power of perceived usefulness decreased, and all variables generated positive nonlinear marginal effects on adoption intention.

**Discussion:**

These findings offer empirical evidence and practical references for optimizing and promoting rural smart mutual-aid care services for older adults in a targeted manner.

## 1 Introduction

In the context of the national strategies of actively addressing population aging and promoting rural revitalization, the aging process in rural China continues to intensify, posing a significant challenge to people's livelihood security and rural governance in the form of rural care for older adults (1). In comparison with the well-established care service system for older adults in urban areas, rural areas are confronted with three key bottlenecks: insufficient care resources for older adults, inadequate professional services, and the erosion of traditional family-based care functions for older adults (2, 3). Conventional home-based care and institutional care models are barely meet the actual care demands of rural older adults (4, 5). As a newly developed and widely promoted care model for older adults adopted to rural areas, mutual-aid care for older adults leverages neighborhood support and rural kinship networks, and features low operational costs and high contextual adaptability (6, 7). Along with the advancement of digital rural construction, intelligent technologies have been gradually deployed in rural care scenarios for older adults (8–10). By integrating digital technology with neighborhood mutual-assistance, intelligent mutual-aid care services for older adults can effectively offset the shortages of rural care resources for older adults and improve the professionalization of care services for older adults (11, 12). This model has become a key approach to addressing the challenges of rural care for older adults, optimizing the rural care system for older adults, and ensuring the quality of life of rural older adults, and thus carries important practical implications and research value.

However, the promotion of smart mutual-aid care services for older adults in rural areas is still at the preliminary stage and faces a variety of practical constraints. Rural older adults generally lack digital literacy and proficiency in operating intelligent devices, and show heterogeneous levels of cognition and acceptance of smart care services for older adults. This has led to low rates of service utilization and diffusion (13, 14). Existing research and practices mainly focus on the construction and optimization of service models, but neglect the micro-level driving mechanisms that influence older adults' acceptance of relevant services. Most existing empirical studies rely on traditional linear models, which fail to adequately account for estimation biases caused by multicollinearity among variables and also fail to capture non-linear relationships between variables (15, 16). As a result, these studies cannot accurately estimate the actual associations of various influencing factors, limiting the explanatory power of existing research conclusions. Accordingly, existing research cannot provide robust empirical support and a solid decision-making foundation for the targeted promotion of smart mutual-aid care services. Therefore, it is theoretically and practically urgent to systematically investigate the determinants of rural older adults' willingness to adopt smart mutual-aid care services, and clarify the internal associations of interaction among variables.

At present, both domestic and international scholars have accumulated substantial research findings on smart care, mutual-aid care, and the acceptance of care services (4, 11, 17). Regarding care models for older adults, researchers have confirmed the applicability of mutual-aid care for older adults in rural contexts and verified that intelligent empowerment can effectively improve the quality and efficiency of care services (18, 19). In terms of service adoption willingness, most existing studies are anchored in the Technology Acceptance Model (TAM) (20, 21) and the Theory of Planned Behavior (TPB) (2, 16, 22, 23). These studies construct research frameworks using core variables including perceived usefulness, perceived ease of use, and social support, and employ multiple linear regression and Structural Equation Modeling (SEM) to test the variable relationships (24, 25). Despite a large number of existing findings, some research gaps remain. First, the majority of current research focuses on urban smart care scenarios, while empirical research targeting rural older adults and the integrated scenario of smart mutual-aid care remains relatively limited. Second, constrained by methodological choices, existing studies over-rely on traditional linear statistical models and overlook the common multicollinearity present in questionnaire survey data, which easily leads to biased model coefficients and statistically insignificant variables. These limitations make it difficult to objectively reflect the independent contribution of each influencing factor. Third, traditional models can only capture linear relationships among variables and fail to account for the underlying non-linear characteristics and their interconnections in the process of service adoption intention formation, thereby resulting in insufficient explanatory power regarding the marginal effects of variables. Overall, existing studies cannot fully and accurately reveal the driving mechanism of rural older adults' willingness to adopt smart mutual-aid care services.

Based on the above research gaps, this study selects rural older adults in Anhui provinces of China as the research subjects and focuses on their willingness to adopt smart mutual-aid older adults care services. In accordance with the TAM model, we set perceived ease of use (PEU), perceived usefulness (PU) and social support (SS) as the core influencing variables, and collect 320 valid samples through questionnaire surveys. Different from prevent studies that merely adopt linear models and SEM, we combine traditional statistical methods with explainable machine learning to construct a comprehensive empirical analysis. Specifically, we first perform descriptive statistics, reliability and validity tests, correlation analysis, and multicollinearity tests to validate data quality. We then apply multiple linear regression to conduct preliminary hypothesis testing. Given that linear models cannot capture potential non-linear effects and interaction effects among variables, this study introduces three tree-based algorithms for comparative modeling. Multiple train-test split strategies and repeated cross-validation schemes are adopted to compare the generalization performance, anti-overfitting ability, and robustness of the three models comprehensively. After confirming Extreme Gradient Boosting (XGBoost) (26, 27) achieves optimal predictive stability, we further calculate its feature importance to quantify the independent predictive contribution of each latent dimension, and utilize Partial Dependence Plots (PDP) to visualize the non-linear marginal association of PEU, PU, and SS with adoption intention.

The contributions of our study are in two aspects. Theoretically, this study integrates explainable machine learning into the research on rural care behaviors for older adults. Specifically, we innovatively combine XGBoost with the classical TAM framework in the specific context of rural smart mutual-aid care, and empirically verify that this integration effectively mitigates multicollinearity in the modified framework. Furthermore, this study proposes a standardized methodological paradigm for TAM-related empirical research. Instead of relying exclusively on linear extensions or regularization adjustments to address high variance inflation factor (VIF) values, we explicitly recommend that when researchers report routine VIF test results for latent constructs, if moderate collinearity is detected between core explanatory variables, tree-based machine learning models can be adopted as complementary interpretive tools to offset the inherent limitations of linear parametric models. This approach enriches the methodological system for research on adoption of smart care intention services, and improves the research framework of the influence mechanism underlying rural smart mutual-aid care for older adults. Practically, this study accurately identifies the core determinants and decision rules that affect the service adoption willingness of rural older adults. The findings of our study can provide actionable implications for local grassroots governments and providers to streamline service interfaces, optimize social support networks, and adapt smart care systems to the heterogeneous and nuanced needs of rural older adults.

## 2 Materials and methods

### 2.1 Research subjects and data collection

This study focused on rural older adults in Anhui Province, aiming to explore their willingness to adopt smart mutual-aid care services and the corresponding influencing factors. Questionnaires were distributed via offline field visits. To balance statistical representativeness and field implementation feasibility, this study employed a three-stage stratified probability sampling strategy targeting rural residents aged 60 and above in Huainan City, Anhui Province, in accordance with standardized survey methodologies (28). The stratified proportional sampling framework covering all 9 county-level administrative regions of Huainan City (Shouxian County, Fengtai County, Tianjia'an District, Datong District, Xiejiaji District, Bagongshan District, Panji District, Maoji Experimental District) was adopted to guarantee sample representativeness.

During field data collection, 336 questionnaires were distributed, and 320 valid responses were recovered, resulting in an effective response rate of 95.24%. This rate exceeds the conventional theoretical threshold for social survey research, thereby ensuring the statistical robustness of the analysis. While this multi-stage sampling design substantially mitigates subjective selection bias relative to purely non-probability sampling methods, potential limitations remain. These include residual time-location substitution bias arising when temporarily absent older participants are replaced with available individuals, as well as the geographic restriction inherent to conducting research in a single prefecture-level municipality. A comprehensive description of the three-stage sampling procedure, village stratification criteria, and detailed derivations of the sample size formula are provided in Supplementary Appendix A.

### 2.2 Questionnaire design and variable definition

#### 2.2.1 Questionnaire design

To ensure all respondents evaluate the same standardized service scenario, we explicitly define the scope and core components of smart mutual-aid care before questionnaire distribution:

The service targets rural older adults in village communities of Huainan, and consists of three core components: (1) intelligent emergency call devices for neighborhood and family assistance; (2) portable smart health monitoring terminals for daily physical examination; (3) village online mutual-aid platform for pension information and help requests.

Before filling out the questionnaire, every interviewee received identical verbal guidance as follows:

“This survey studies rural smart mutual-aid care, including emergency call equipment, home health monitors and village online mutual aid tools. All questions refer to these three types of services only. All answers are anonymous and only used for academic research; we will provide one-on-one assistance if you have trouble understanding any items.”

We have placed the specific questionnaire content in Supplementary Appendix B.

#### 2.2.2 Variable definition

The questionnaire was developed based on the TAM model and well-established scales in the field of relevant care research (29). All observed items and detailed measurement indicators of the four latent variables are summarized in Table 1.

**Table 1:** Measurement items of latent variables.

Latent variable	Abbreviation	Item	Observed item
Perceived ease of use	PEU	PEU1	I think the operation of smart mutual-aid care services is simple.
Perceived ease of use	PEU	PEU2	I can quickly learn to use smart mutual-aid care services.
Perceived ease of use	PEU	PEU3	It is not difficult for me to operate the smart devices supporting care services.
Perceived usefulness	PU	PU1	Smart mutual-aid care services can help me solve daily pension problems.
Perceived usefulness	PU	PU2	Using these services can effectively improve my quality of life.
Perceived usefulness	PU	PU3	Smart mutual-aid care services have practical value for rural older adults.
Social support	SS	SS1	My family members will guide me to use smart mutual-aid care services.
Social support	SS	SS2	Neighbors and friends will offer assistance when I encounter operational difficulties.
Social support	SS	SS3	Village or community staff provide help for me to use relevant smart care services.
Adoption intention	BI	BI1	I am willing to use smart mutual-aid care services actively.
Adoption intention	BI	BI2	I plan to continue using these services in the future.
Adoption intention	BI	BI3	I would recommend these services to other people around me.

A 5-point Likert scale was adopted for all items: 1 = strongly disagree, 2 = disagree, 3 = neutral, 4 = agree, 5 = strongly agree (30). A higher score represents a higher level of respondents' recognition. This study included four latent variables, consisting of three exogenous independent variables and one endogenous dependent variable.

- PEU: An exogenous variable, referring to rural older adults' subjective perception of the operational difficulty of smart mutual-aid care services (31).
- PU: An exogenous variable, representing rural older adults' recognition of the practical value of the services (32).
- SS: An exogenous variable, referring to external assistance and emotional support from family members, neighbors and communities during service usage (33).
- Adoption Intention (BI): An endogenous dependent variable, indicating rural older adults' subjective tendency to use smart mutual-aid care services (34).

### 2.3 Data analysis methods

All data processing and analysis were implemented using Python. Preliminary tests verified moderate multicollinearity among independent variables, which would bias the results of SEM model (24). While SEM is widely adopted to estimate linear structural pathways and correct for measurement errors, moderate multicollinearity among latent constructs can substantially bias parameter estimation in covariance-based SEM (CB-SEM). Existing literature indicates that even moderate multicollinearity [even with a variance inflation factor (VIF) < 20] significantly inflates the standard errors of path coefficients, may reverse the sign of estimated coefficients, and reduces statistical power, ultimately resulting in unreliable significance testing for driving factors. Furthermore, existing common remedial strategies cannot fully address the limitations of CB-SEM; for example, increasing sample size only reduces sampling variance but does not eliminate structural correlations between latent variables. Additionally, regularized linear methods including ridge regression and partial least squares SEM (PLS-SEM) can compress coefficient variance induced by collinearity. However, these methods are still constrained by strict linearity assumptions and cannot identify non-linear threshold effects or high-order interaction effects.

Consequently, the primary reasons for selecting the XGBoost model are its non-parametric flexibility and superior predictive performance. Unlike conventional linear frameworks, which require strict distributional assumptions, XGBoost does not depend on such restrictive assumptions and can automatically capture complex non-linear relationships and multivariate interaction effects that are omitted by traditional linear specifications. Furthermore, in contrast to the constant marginal effects estimated by traditional SEM, XGBoost dynamically quantifies feature importance via iterative tree-based ensemble learning.

#### 2.3.1 Basic statistical tests

First, descriptive statistics were used to analyze data distribution characteristics. Skewness and kurtosis were applied to judge whether the data followed a normal distribution. Then, Cronbach's α coefficient was used for reliability test, and Kaiser-Meyer-Olkin (KMO) test together with Bartlett's test of sphericity and Exploratory Factor Analysis (EFA) were adopted for validity test (35, 36). The unified judgment criteria for the above tests are shown in Table 2.

**Table 2:** Judgment criteria for basic statistical tests.

Test type	Evaluation index	Judgment standard
Normality test	Skewness and Kurtosis	Absolute value < 2, approximately normal distribution
Reliability test	Cronbach's α	*α* > 0.8: excellent reliability
		0.7 < α ≤ 0.8: acceptable reliability
Suitability for EFA	KMO value	KMO > 0.8: very suitable for factor analysis
Suitability for EFA	Bartlett's test	*p* < 0.05: correlation exists among variables
Convergent validity	Factor loading	Loading >0.45: qualified; loading >0.5: excellent

(1) Descriptive statistics

Mean value and standard deviation (SD) were calculated to describe the central tendency and dispersion of data, as shown in Equations 1 and 2.


$$\begin{array}{l} \bar{x}=\frac{1}{n}\overset{n}{\underset{i=1}{\sum }}x_{i} \end{array}$$ (1)

$$\begin{array}{l} S=\sqrt{\frac{1}{n-1}\overset{n}{\underset{i=1}{\sum }}{\left(x_{i}-\bar{x}\right)}^{2}} \end{array}$$ (2)

where *n* is the sample size, *x*_*i*_ is the value of the *i*-th sample, $\bar{x}$ represents the mean value of the variable, and *S* denotes the standard deviation.

(2) Reliability and validity test

Cronbach's *α* coefficient was used to evaluate the internal consistency of the questionnaire. A value of *α* > 0.8 indicates excellent reliability. The formula is shown as Equation 3.


$$\begin{array}{l} \alpha =\frac{k}{k-1}\left(1-\frac{\overset{k}{\underset{j=1}{\sum }}S_{j}^{2}}{S_{t}^{2}}\right) \end{array}$$ (3)

where *k* is the number of observed items for a single latent variable, $S_{j}^{2}$ represents the variance of the *j*-th item, and $S_{t}^{2}$ denotes the total variance of all items belonging to the same latent variable.

The KMO test and Bartlett's test of sphericity were used to judge the feasibility of factor analysis. EFA was further conducted to assess convergent and structural validity. The dataset is suitable for factor analysis when KMO > 0.8 and *p* < 0.05 for Bartlett's test. A factor loading greater than 0.5 indicates acceptable convergent validity (37).

#### 2.3.2 Traditional linear analysis

This section included Pearson correlation analysis, multicollinearity test and multiple linear regression analysis (1, 6, 36). The judgment criteria for correlation strength, multicollinearity and regression model significance are presented in Table 3.

**Table 3:** Judgment criteria for correlation, multicollinearity and regression.

Test type	Evaluation index	Judgment standard
Correlation strength	Pearson correlation coefficient *r*	*R* > 0.8: high correlation
		0.5 < *r* < 0.8: moderate correlation
Multicollinearity	Variance Inflation Factor (VIF)	VIF < 10: no severe multicollinearity
		10 ≤ VIF < 100: moderate multicollinearity
Regression significance	*p*-value of coefficient	*p* < 0.05: statistically significant
Residual autocorrelation	Durbin-Watson	DW ≈ 2: no serial autocorrelation

First, we employed the Pearson correlation coefficient to measure the strength and direction of linear relationships between variables, as shown in Equation 4.


$$\begin{array}{l} r_{xy}=\frac{\overset{n}{\underset{i=1}{\sum }}\left(x_{i}-\bar{x}\right)\left(y_{i}-ȳ\right)}{\sqrt{\overset{n}{\underset{i=1}{\sum }}{\left(x_{i}-\bar{x}\right)}^{2}}\sqrt{\overset{n}{\underset{i=1}{\sum }}{\left(y_{i}-ȳ\right)}^{2}}} \end{array}$$ (4)

where *r*_*xy*_ is the Pearson correlation coefficient between variable *x* and *y*, ranging from −1 to 1; $\bar{x}$ and *ȳ* represent the mean values of the two variables, respectively.

Second, we used the VIF to detect multicollinearity, as shown in Equation 5.


$$\begin{array}{l} \text{VI}{\text{F}}_{j}=\frac{1}{1-R_{j}^{2}} \end{array}$$ (5)

where VIF_*j*_ is the VIF of the *j*-th independent variable, $R_{j}^{2}$ is the coefficient of determination of the regression model using the *j*-th variable as the dependent variable and other variables as predictors. In addition, VIF < 10 means no severe multicollinearity, and 10 ≤ VIF < 100 represents moderate multicollinearity.

Third, taking BI as the dependent variable and PEU, PU, SS as independent variables, the multiple linear regression model was constructed as Equation 6.


$$\begin{array}{l} \text{BI}=\beta_{0}+\beta_{1}\text{PEU}+\beta_{2}\text{PU}+\beta_{3}\text{SS}+\varepsilon \end{array}$$ (6)

where *β*_0_ is the intercept term, *β*_1_, *β*_2_, *β*_3_ are the regression coefficients, respectively; ε denotes the random error term. The *p*-value of each coefficient was used to test statistical significance.

#### 2.3.3 XGBoost and PDP analysis

XGBoost model was adopted to avoid multicollinearity bias and capture non-linear relationships (38). The model performance was evaluated by *R*^2^, Root Mean Squared Error (RMSE) and Mean Absolute Error (MAE), and the judgment criteria are shown in Table 4. PDP was used to analyze marginal effects.

**Table 4:** Evaluation criteria for XGBoost model.

Evaluation index	Meaning	Judgment standard
Coefficient of determination (*R*^2^)	Explanatory power of model	R2 closer to 1: better fitting performance
RMSE	Prediction error	Smaller value: higher prediction accuracy
MAE	Average deviation	Smaller value: higher prediction accuracy

(1) XGBoost regression model

XGBoost, an ensemble tree model robust to multicollinearity, was used for regression modeling to capture non-linear relationships. The final prediction is the sum of outputs from multiple decision trees, as illustrated in Equation 7.


$$\begin{array}{l} {ŷ}_{i}=\overset{T}{\underset{t=1}{\sum }}f_{t}\left(x_{i}\right),\;f_{t}\in F \end{array}$$ (7)

where *ŷ*_*i*_ is the predicted value of the *i*-th sample, *T* is the total number of decision trees, *f*_*t*_ (*x*_*i*_) denotes the output of the *t*-th tree, and F is the function space of all decision trees.

The objective function of XGBoost consists of loss function and regularization term, which balances model accuracy and overfitting prevention. The objective function is shown in Equation 8.


$$\begin{array}{l} \text{Objective}=\overset{n}{\underset{i=1}{\sum }}l\;\left(y_{i},{ŷ}_{i}\right)+\overset{T}{\underset{t=1}{\sum }}\Omega \;\left(f_{t}\right) \end{array}$$ (8)

where *l* (*y*_*i*_, *ŷ*_*i*_) is the squared loss function for regression tasks, and Ω (*f*_*t*_) is the regularization term for controlling tree complexity.

The dataset was split into a training set and a test set at a ratio of 7:3 in this study. Coefficient of determination *R*^2^, RMSE and MAE were used to evaluate model performance. Feature importance was extracted to quantify the independent contribution of each variable.

(2) PDP

PDP was applied to visualize the marginal effect of a single variable on the dependent variable (39), as shown in Equation 9.


$$\begin{array}{l} \text{PD}\left(x_{s}\right)={𝔼}_{x_{c}}\left[\hat{f}\left(x_{s},x_{c}\right)\right] \end{array}$$ (9)

where *x*_*s*_ is the target independent variable, *x*_*c*_ is the other covariates, $\hat{f}\left(\cdot \right)$ denotes the trained XGBoost model, and PD(*x*_*s*_) represents the partial dependence value, reflecting the average marginal trend of the target variable.

## 3 Results and discussion

### 3.1 Descriptive statistics and reliability and validity tests

**Figure 1:**
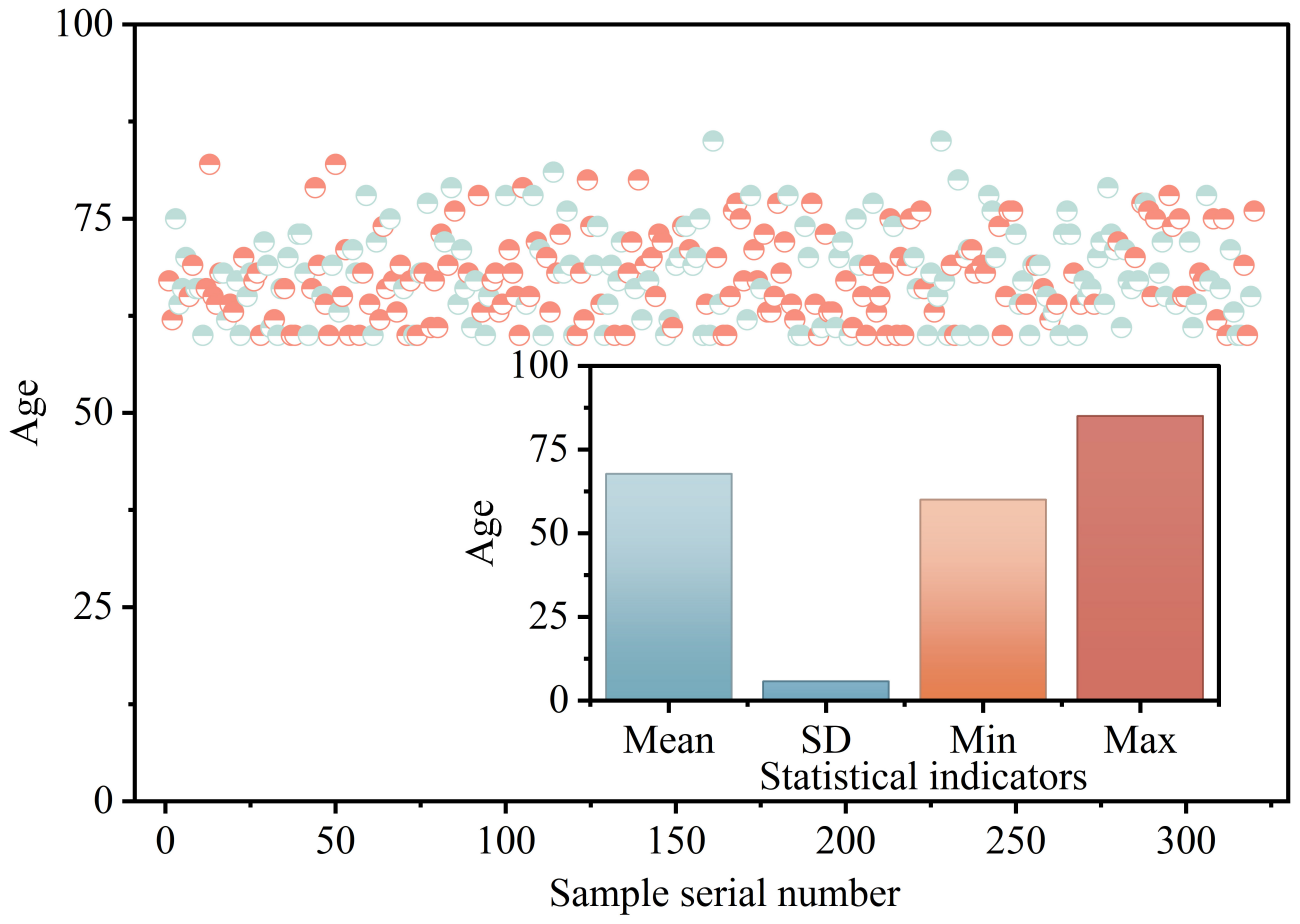
Demographic characteristics of rural older adults.

A total of 320 valid questionnaires were collected in this study (Figure 1). The average age of participants was 67.73 years old (SD = 5.77), with the age range from 60 to 85 years old. The demographic characteristics indicated that the samples were representative of rural older adults in Anhui Province.

**Figure 2:**
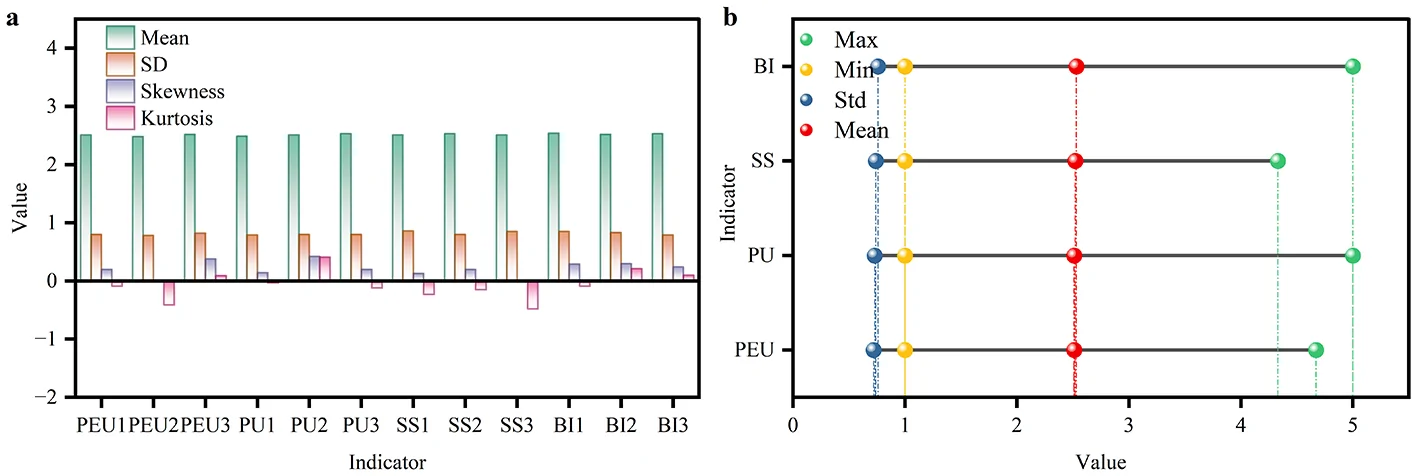
Descriptive statistics of all observed items and latent variables. **(a)** Values of mean, SD, skewness, and kurtosis for all indicators. **(b)** Scores of four latent variables including mean, SD, maximum, and minimum.

Moreover, descriptive statistics, including the means, standard deviations, skewness, and kurtosis of all individual observed items and four latent variables, are summarized in Figure 2. For all measurement items, the absolute values of skewness and kurtosis are all below 2, confirming that the sample data approximately follows a univariate normal distribution (Figure 2a). This normality characteristic meets the fundamental statistical prerequisite for subsequent reliability and validity testing, correlation analysis, and regression modeling. Furthermore, Figure 2b presents the aggregated descriptive statistics of the four core latent variables. The descriptive results of the latent variables indicate that the mean score of PEU is 2.51 (SD = 0.72, range: 1.00–4.67); PU also has a mean of 2.51 (SD = 0.73, range: 1.00–5.00); SS yields a mean value of 2.52 (SD = 0.74, range: 1.00–4.33); and BI has the highest mean score of 2.53 (SD = 0.76, range: 1.00–5.00). With 3 set as the midpoint of the full measurement scale, the average scores of all four latent variables are significantly lower than the medium threshold of 3. Specifically, rural older adults reported low perceived ease of use regarding smart mutual-aid care services, which indicates that most respondents perceive these intelligent care services as cumbersome and difficult to operate. Meanwhile, respondents only held neutral-to-low recognition of the practical benefits delivered by these services, which is consistent with the low mean score of PU. Regarding social support, rural older adults receive only moderate external support for adopting these smart care services. Furthermore, their overall willingness to use smart mutual-aid care services remains relatively low. Taken together, the high operational complexity of intelligent senior care services is the primary barrier hindering the acceptance and adoption of such services among rural older adults (13, 17).

Cronbach's *α* coefficient was adopted to examine the internal consistency reliability of the questionnaire. As shown in Table 5, the Cronbach's *α* values of PEU, PU, SS and BI were 0.8767, 0.9003, 0.8542, and 0.9080 respectively. All coefficients were greater than 0.8, which proved that each dimension of the questionnaire had excellent internal consistency. The reliability of the scale fully satisfied the requirements for subsequent empirical analysis.

**Table 5:** Results of reliability test.

Latent variable	Number of items	Cronbach's α
PEU	3	0.8767
PU	3	0.9003
SS	3	0.8542
BI	3	0.9080

KMO test and Bartlett's test of sphericity were conducted to verify whether the dataset was suitable for EFA. The results are shown in Table 6: the KMO value was 0.8929, and the Chi-square value of Bartlett's test was 2557.0135 with *p* < 0.001. It indicated significant correlations among observed items, and the dataset was appropriate for factor analysis.

**Table 6:** Results of KMO test and Bartlett's test.

Test item	Value
KMO value	0.8929
Chi-square value of Bartlett's test	2557.0135
*P*-value of Bartlett's test	0.000

Four common factors were extracted with maximum variance rotation in EFA. The results are summarized in Table 7. The cumulative variance explained rate of the four factors reached 81.58%, which could interpret most information of the original data. All factor loadings of observed items were higher than 0.45, and most loadings exceeded 0.6. The item distribution was highly consistent with the theoretical dimension division. In general, the questionnaire possessed good convergent validity and structural validity.

**Table 7:** Results of exploratory factor analysis.

Common factor	Value	Variance explained rate (%)	Cumulative variance explained rate (%)
Factor 1	2.5433	21.19	21.19
Factor 2	2.3889	19.91	41.10
Factor 3	2.4776	20.63	61.74
Factor 4	2.3815	19.85	81.58

### 3.2 Correlation and multicollinearity analysis

Pearson correlation analysis was performed to examine the linear relationships among PEU, PU, SS, and BI, with correlation coefficients reported in Table 8. The results indicate that all core variables are significantly positively correlated with one another (*p* < 0.001). Specifically, the correlation coefficient between PEU and PU is 0.594, indicating a moderate linear association between these two perceptual dimensions. The correlations between PEU and BI (*r* = 0.633) and between PU and BI (*r* = 0.644) are relatively strong. By contrast, SS exhibits a weak correlation with both PEU (*r* = 0.166) and PU (*r* = 0.166), and a moderate correlation with BI (*r* = 0.357). Overall, all latent variables covary in the same direction among rural older adults in Anhui Province.

**Table 8:** Significance results for variables.

Latent variable	PEU	PU	SS	BI
PEU	1.0	0.0	0.0	0.0
PU	0.0	1.0	0.0	0.0
SS	0.0	0.0	1.0	0.0
BI	0.0	0.0	0.0	1.0

**Figure 3:**
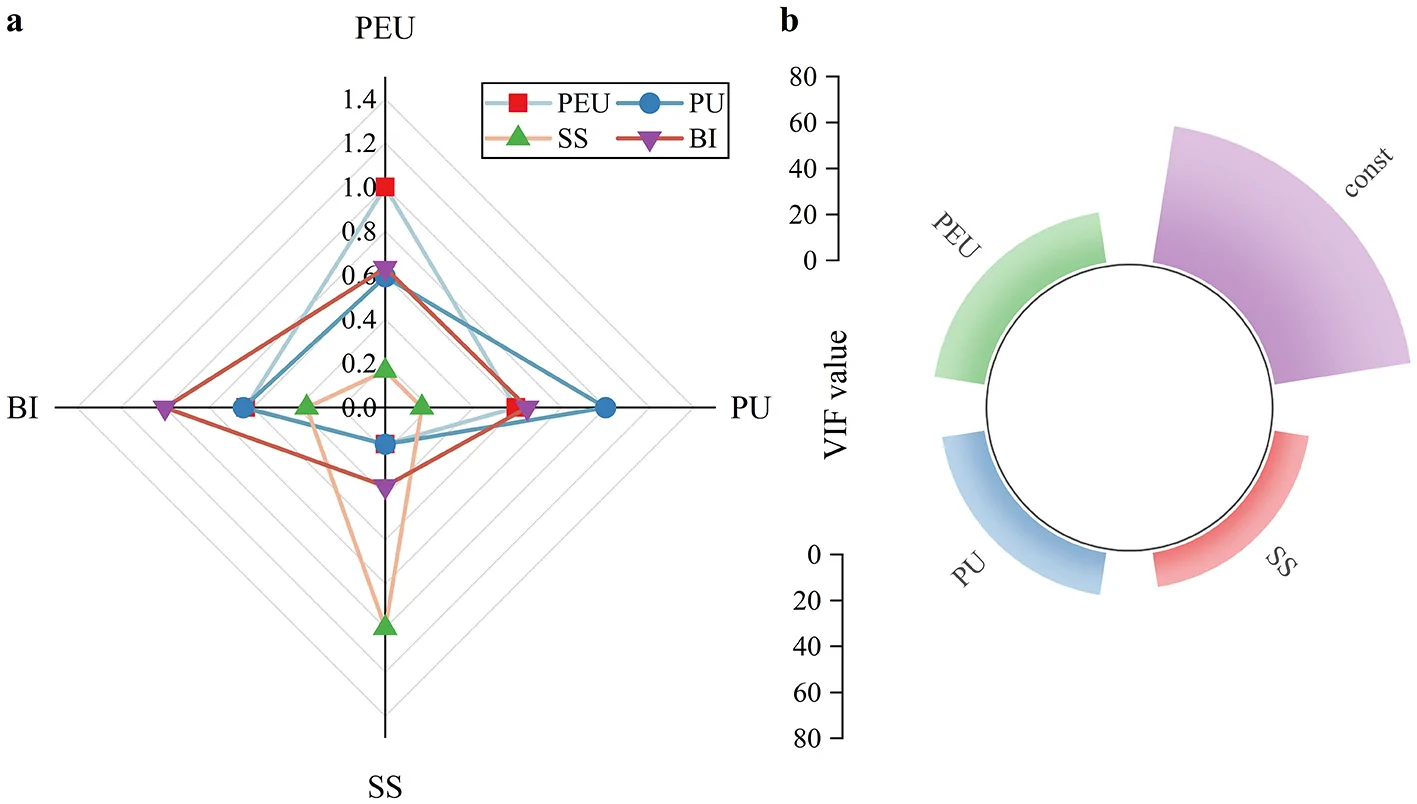
Results of correlation and multicollinearity analysis. **(a)** Results of correlation coefficients in Pearson correlation analysis. **(b)** VIF value of independent variables including PEU, PU, and SS.

To further quantify multicollinearity among the independent variables PEU, PU, and SS, variance inflation factor (VIF) values were calculated and presented in Figure 3b. Excluding the intercept term, whose VIF value has no statistical significance, the VIF values of PEU, PU, and SS are 21.96, 18.61, and 15.09 respectively, which confirms the presence of linear collinearity among the predictors. Furthermore, the moderate zero-order correlation between PEU and PU (*r* = 0.594) (Figure 3a) can introduce potential estimation bias to conventional linear models including OLS regression and SEM. Linear modeling frameworks rely on the strict assumption of independence among explanatory variables, and moderate multicollinearity and pairwise construct correlation will inflate standard errors, destabilize path coefficient estimates, and prevent accurate separation of the independent predictive effects of PEU and PU (1, 40). Given that tree-based machine learning algorithms are robust to mild and moderate linear correlations among variables and can additionally capture unobserved non-linearities and interaction effects, the necessity of adopting these algorithms in this study is justified.

### 3.3 Results of multiple linear regression

Based on the above correlation and multicollinearity tests, this study constructed an ordinary least squares (OLS) multiple regression model (41, 42) with BI as the dependent variable and PEU, PU and SS as independent variables.

Table 9 presents the OLS regression results obtained with heteroskedasticity-robust HC3 standard errors. Two model specifications are established: Model 1 includes only the three core theoretical dimensions (PEU, PU, SS), while Model 2 further incorporates six control variables (Gender, Age, Edu, Health, Living, Service) to alleviate omitted variable bias. After adding the control variables, the adjusted *R*^2^ increases from 0.560 to 0.630, which indicates that individual characteristic variables contribute substantially to explaining the adoption intention of the rural older adults. PEU, PU, and SS are significantly positively associated with adoption intention at the 1% level in both models. PU has the largest coefficient and thus acts as the dominant driving factor of adoption intention. Among the control variables, Age, Living arrangement (Living) and past service experience (Service) are statistically significantly positively associated with adoption intention, while Gender, education level (Education) and physical health (Health) do not exhibit a significant predictive effect. Notably, the condition number of Model 2 reaches 962, indicating moderate multicollinearity among the core latent constructs, which may compromise the accuracy of linear estimation for both CB-SEM and OLS. This limitation of linear models provides the motivation for adopting the XGBoost algorithm, which is robust to collinearity and capable of capturing unobservable non-linear relationships.

**Table 9:** OLS regression results on rural older adult's service adoption intention.

Variables	Model 1 (no controls)	Model 2 (with all controls)
Intercept	−0.0464 (0.134)	−2.0360^***^ (0.442)
PEU	0.3835^***^ (0.052)	0.3895^***^ (0.051)
PU	0.4054^***^ (0.048)	0.4331^***^ (0.045)
SS	0.2381^***^ (0.038)	0.2489^***^ (0.038)
Gender	–	−0.0679 (0.053)
Age	–	0.0237^***^ (0.006)
Education	–	0.0355 (0.026)
Health	–	0.0022 (0.035)
Living	–	0.1710^**^ (0.066)
Service	–	0.2867^***^ (0.055)
Adj.R^2^	0.560	0.630
Condition number	412	962
*N*	320	320

Values in parentheses are heteroskedasticity robust standard errors. ^***^p < 0.001, ^**^p < 0.05, ^*^p < 0.1.

### 3.4 Results of XGBoost and partial dependence

#### 3.4.1 Performance comparison of different machine learning algorithms

Given the presence of moderate multicollinearity among the core explanatory variables, and the inherent limitations of conventional linear OLS models in capturing non-linear threshold effects and interaction effects, this study further introduces three tree-based ensemble algorithms—Random Forest, LightGBM, and XGBoost—to construct comparative predictive models (43). Multiple train-test split schemes and cross-validation strategies are adopted to comprehensively evaluate the generalization performance, prediction accuracy, and anti-overfitting capability of the constructed models.

**Figure 4:**
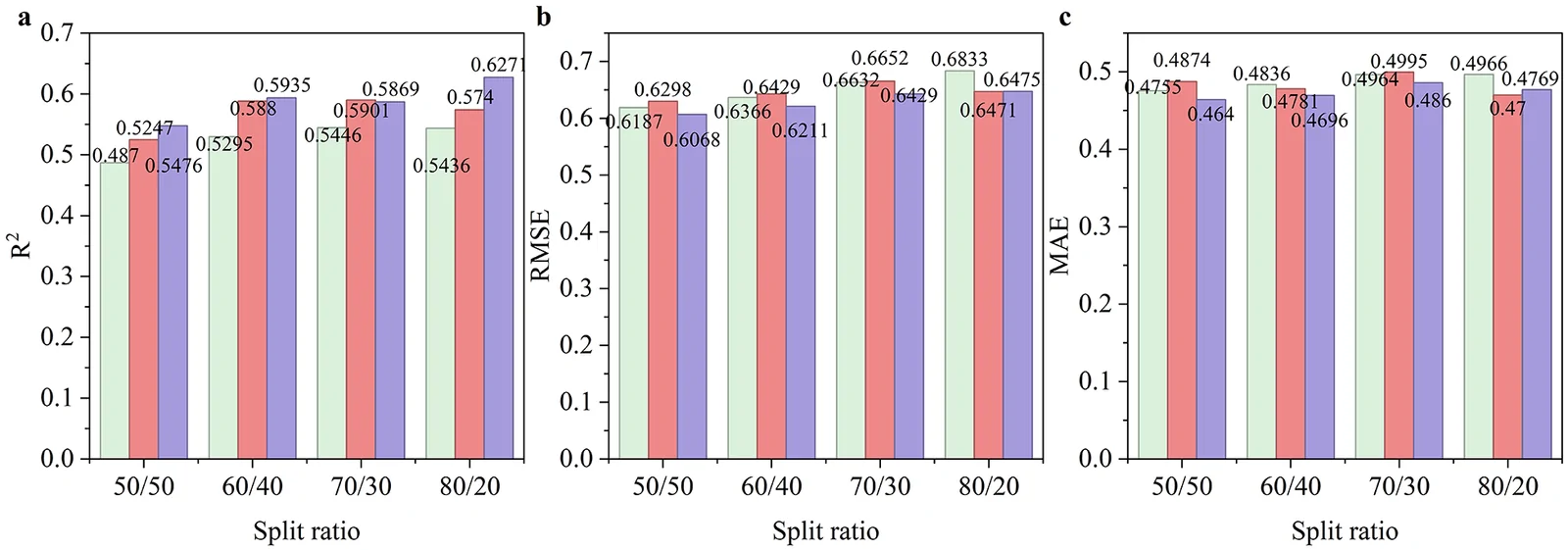
Model performance with single train-test segmentation. **(a)** Coefficient of determination (*R*^2^); **(b)** Root mean square error (RMSE); **(c)** Mean absolute error (MAE).

To preliminarily compare the generalization performance of the Random Forest, LightGBM, and XGBoost algorithms, this study adopts four fixed train-test splitting ratios (50/50, 60/40, 70/30, 80/20), and selects Train *R*^2^, Test *R*^2^, Train RMSE, Test RMSE, Train MAE, and Test MAE as evaluation metrics. Figure 4 presents the single-split prediction results of the three machine learning models. Across all splitting schemes, XGBoost achieves the highest Test R^2^ and the lowest Test RMSE and Test MAE, indicating that it has superior out-of-sample predictive capability for the adoption intention of rural older adults. In contrast, LightGBM exhibits severe overfitting: its Train *R*^2^ ranges from 0.8221 to 0.8401, while the corresponding Test *R*^2^ drops sharply, resulting in a large performance gap between the training set and the testing set. Random Forest suffers from underfitting, which is reflected in its low Train *R*^2^ across all groups. Overall, XGBoost achieves a more balanced trade-off between fitting ability and generalization performance among the three algorithms.

To eliminate random bias introduced by single data partitioning and verify the stability of model inference, four cross-validation strategies were further implemented: standard 5-fold CV, standard 10-fold CV, 5-time repeated 5-fold CV, and 3-time repeated 10-fold CV. All cross-validation results are summarized in Table 10, where model performance is reported as Mean ± Standard Deviation. Consistent with the results from single-split experiments, LightGBM exhibits the lowest average Test *R*^2^ and larger standard deviations across all validation settings, indicating poor generalization ability and high result volatility. Random Forest achieves moderate prediction performance, while XGBoost obtains the optimal overall performance: it retains the highest average Test *R*^2^ and the lowest Test RMSE/Test MAE in most validation schemes, with relatively low standard deviations of all evaluation metrics. These results confirm that XGBoost delivers higher prediction accuracy and greater robustness against random data partitioning.

**Table 10:** Cross-validation performance of three machine learning models.

Type	Model	Test *R*^2^ (Mean ±Std)	Test RMSE (Mean ±Std)	Test MAE (Mean ±Std)
5-fold CV	RandomForest	0.4056 ± 0.0992	0.5538 ± 0.0914	0.4135 ± 0.0589
5-fold CV	LightGBM	0.3466 ± 0.1103	0.5766 ± 0.0687	0.4391 ± 0.0494
5-fold CV	XGBoost	0.4012 ± 0.0928	0.5544 ± 0.0786	0.4140 ± 0.0525
10-fold CV	RandomForest	0.4199 ± 0.1156	0.5403 ± 0.0934	0.4051 ± 0.0624
10-fold CV	LightGBM	0.3395 ± 0.1862	0.5688 ± 0.0899	0.4317 ± 0.0635
10-fold CV	XGBoost	0.4100 ± 0.1530	0.5402 ± 0.0896	0.4038 ± 0.0575
Repeated 5-fold ( × 5)	RandomForest	0.4084 ± 0.1180	0.5552 ± 0.0809	0.4111 ± 0.0432
Repeated 5-Fold ( × 5)	LightGBM	0.3540 ± 0.1116	0.5793 ± 0.0688	0.4325 ± 0.0405
Repeated 5-fold ( × 5)	XGBoost	0.4063 ± 0.1110	0.5560 ± 0.0769	0.4123 ± 0.0425
Repeated 10-fold ( × 3)	RandomForest	0.4152 ± 0.1255	0.5417 ± 0.1101	0.4049 ± 0.0615
Repeated 10-fold ( × 3)	LightGBM	0.3589 ± 0.1544	0.5639 ± 0.1053	0.4222 ± 0.0635
Repeated 10-fold ( × 3)	XGBoost	0.4134 ± 0.1419	0.5404 ± 0.1096	0.4044 ± 0.0593

Overall, XGBoost outperforms alternative tree-based models in balancing prediction accuracy, generalization stability, and collinearity resistance. Accordingly, this study adopts XGBoost as the core interpretable machine learning tool to further explore the non-linear correlations between research variables.

#### 3.4.2 Feature importance scores of three independent variables in XGBoost model

**Figure 5:**
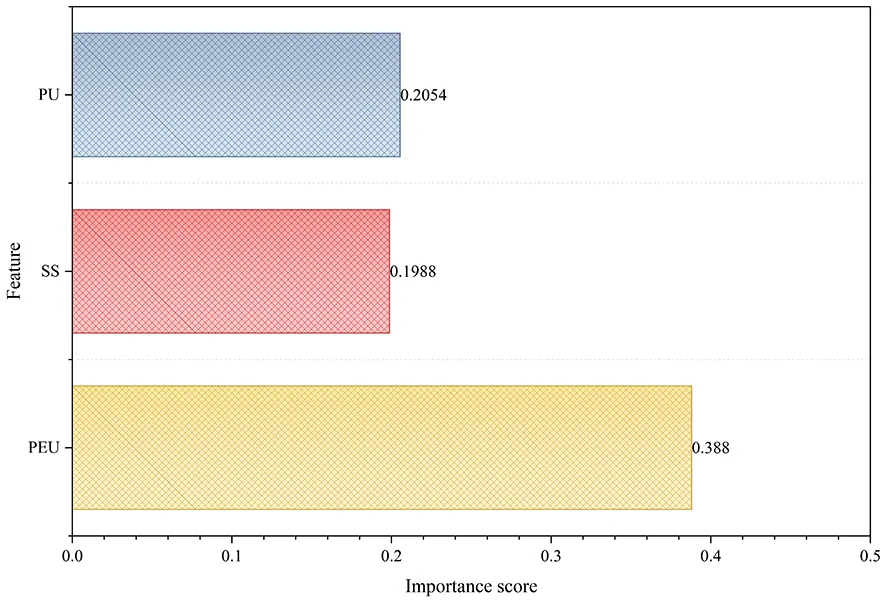
Feature importance score of three independent variables.

The feature importance scores of three independent variables are presented in Figure 5. The ranking from high to low is: PEU (0.388), PU (0.2054), SS (0.1988). This result partially differs from Davis's (20) classic TAM, which generally regards perceived usefulness as the dominant predictor of technology adoption. In the context of rural groups for older adults with limited digital skills, PEU carries the highest predictive weight, nearly 1.9 times that of PU. This demonstrates that rural older adults prioritize the ease of operation of smart mutual-aid care services over functional practical benefits such as daily care, health monitoring and emergency assistance. The feature importance of SS is almost equivalent to that of PU, implying that social networks consisting of family opinions, neighbor referrals and village committee publicity exert a comparable facilitating effect on seniors' adoption willingness, matching the interpersonal features of rural acquaintance societies.

#### 3.4.3 Results of partial dependence analysis

**Figure 6:**
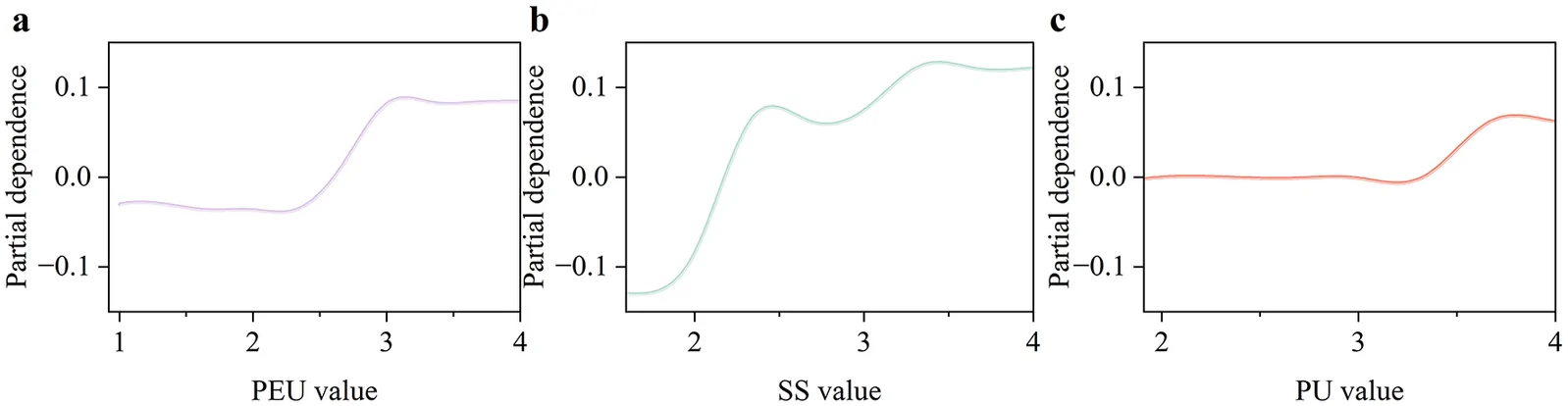
Partial dependence results of all variables on adoption intention. **(a)** PEU. **(b)** SS. **(c)** PU.

Partial Dependence Analysis further reveals the non-linear association patterns of the three factors with acceptance intention (Figure 6). For PEU, the partial dependence curve exhibits an S-shaped growth pattern. When PEU < 2.5, it is negatively associated with users' acceptance intention (PD < 0), indicating that the acceptance willingness of older adults users toward smart care services decreases significantly when they perceive the operation to be overly complex. Once PEU exceeds 2.5, the association rapidly becomes positive and stabilizes at approximately 0.4 for the partial derivative. This indicates that after crossing the PEU threshold, incremental improvements to usability yield diminishing marginal returns for users' acceptance intention. This finding suggests that age-friendly interface optimization should prioritize removing basic operational barriers rather than pursuing excessive refinement. For SS, the partial dependence curve shows a stepwise upward trend. When SS ranges from 1.0 to 2.0, social support has a significantly negative effect on acceptance intention (PD ≈ −0.4), indicating that older adults with insufficient social support have substantially lower acceptance willingness. When SS exceeds 2.5, the association becomes positive and continues to increase before plateauing at SS ≈ 3.5 (PD ≈ 0.4). This confirms that social support has a clear critical threshold, only when support from families, communities, and peer groups reaches or exceeds a moderate level can it be effectively converted into acceptance intention. For PU, the partial dependence curve follows an exponential growth pattern, and has the steepest slope among the three variables. PU has a strong negative association with acceptance intention when it ranges from 1.0 to 2.5 (PD ≈ −0.4). However, once PU exceeds 3.0, the positive association increases rapidly and plateaus at PU ≈ 4.0 (PD ≈ 0.6). This characteristic of a sharp jump verifies that PU is the core driving factor: once older adults recognize the practical value of smart mutual-aid care services, such as telemedicine, one-click emergency calls, and intelligent positioning, their acceptance intention will achieve a qualitative increase.

## 4 Conclusion and policy implications

### 4.1 Conclusions

This study investigates the adoption intention of smart mutual-aid care services among rural older adults in Anhui Province, using an extended TAM framework and a methodological approach that combines OLS regression with the XGBoost machine learning algorithm. The main conclusions are summarized as follows. First, the overall adoption intention of the surveyed rural older adults remains low, with PEU being particularly weak. This indicates that high operational complexity is the primary psychological and practical barrier hindering the penetration of intelligent senior care services in rural areas. Furthermore, OLS encounter estimation limitations due to moderate collinearity among perceptual variables, while the XGBoost algorithm demonstrates superior robustness and predictive accuracy, successfully uncover complex non-linear influencing mechanisms. In addition, diverging from the classic TAM, which prioritizes PU, feature importance analysis reveals that PEU is the dominant predictor of adoption intention. Therefore, for digitally vulnerable rural older adults, operational accessibility outweighs functional utility. Finally, PEU exhibits an S-shaped threshold effect, where its marginal effect stabilizes after PEU exceeds 2.5; SS must cross a moderate threshold (above 2.5) to exert a positive motivating effect; PU follows an exponential growth trajectory, with a qualitative shift in its influence once PU exceeds 3.0.

The predominance of PEU over PU in driving adoption intention among rural older adults can be attributed to three interrelated mechanisms. First, the digital literacy deficit prevalent among rural older adults cohorts (mean age = 67.73 years) fundamentally alters the relative salience of perceived ease-of-use vs. perceived usefulness. Unlike urban populations, rural older adults face substantial cognitive and motor barriers, rendering operational simplicity a prerequisite for technology adoption, rather than a complement to perceived usefulness. Second, the trust deficit in technology-mediated care amplifies the weight of PEU. When the operational process is perceived as opaque, uncertainty escalates into active resistance to adoption, whereas intuitive interfaces provide transparent, controllable interaction experiences that reduce such uncertainty. Third, the socially embedded nature of technology adoption in rural contexts elevates the role of SS to a magnitude near-equivalent to that of PU. Operating within a community of acquaintances, informal social networks—including adult children and village committees—reframe technology use from individual risk-taking into a socially endorsed normative behavior.

Furthermore, the non-linear threshold effects identified via partial dependence analysis elucidate the cognitive-behavioral transition mechanisms underlying technology adoption decisions. The negative partial dependence of PEU at values below 2.5 corresponds to a frustration zone, where operational complexity exceeds individuals' self-efficacy thresholds and induces technology-related anxiety. The abrupt positive shift in the partial dependence of performance expectancy at the 2.5 threshold aligns with the minimum level of social scaffolding required to sustain behavioral trials and overcome initial adoption barriers. Finally, beyond the 3.0 threshold, the partial dependence of PU exhibits exponential growth. Specifically, sustained behavioral motivation can only be derived from perceived usefulness when rural older adults explicitly associate smart services with life-critical benefits such as emergency response and fall detection.

### 4.2 Policy implications

Combined with the research conclusions and current status of rural care services in Anhui Province, this study proposes targeted policy recommendations and actionable optimization measures from three dimensions aligned with the core influencing factors.

(1) Optimize service design and reduce operational barriers. As PEU is the most critical factor affecting service adoption, relevant authorities should prioritize age-friendly transformation of smart care products and platforms (31). It is recommended to simplify operation interfaces, remove redundant functional configurations, and adopt large-font layouts, voice navigation, and one-click operation modes suitable for rural older adults with limited digital literacy (44, 45). Meanwhile, offline training courses should be organized at village and community levels to equip older adults with basic operational skills, fundamentally lowering the adoption threshold of relevant services.

(2) Improve the rural social support system and inherit mutual-aid culture. Rural areas of Anhui Province have a long-standing tradition of neighborhood mutual assistance. Governments and village committees should fully leverage the value of rural social support networks (46). On the one hand, encourage family members, neighbors, and community volunteers to provide targeted guidance and assistance for older adults accessing smart older adult care services. On the other hand, establish rural mutual-aid volunteer teams to form a long-term support mechanism, giving full play to the auxiliary role of social support (47).

(3) Refine service functions and enhance practical value. Although the independent contribution of PU is relatively low due to the existence of multicollinearity, rural older adults generally recognize the practical value of smart mutual-aid care services (48). Service providers should further optimize service content in accordance with the actual care demands of rural older adults, integrate functions including daily living assistance, health management and spiritual comfort, and continuously improve the practicality and attractiveness of services (49).

(4) Facilitate the integrated development of digital rural construction and rural care. Leveraging the development of digital villages as an entry point, further integration of digital technology and rural mutual-aid care is required. It is critical to develop localized smart mutual-aid care models that fit the characteristics of the rural population and regional culture, address weaknesses in rural care service provision, and achieve the long-term sustainable development of rural smart care services.

### 4.3 Limitations and future directions

This study still has some limitations. First, regarding sample representativeness and external generalizability, our dataset was limited to rural areas of Anhui Province. Although rural Anhui is a representative microcosm of central China in terms of population aging rate and economic development level, regional disparities in digital infrastructure and local government fiscal capacity between eastern and western China may restrict the direct generalizability of our findings. Future research should expand the spatial sampling frame to cover eastern, central, and western provinces, and use standardized macro indicators such as local GDP per capita and rural Internet penetration rate to benchmark regional variations. Second, in terms of measurement and methodological constraints, because all latent constructs were measured via self-reported questionnaires administered to the same respondents at a single time point, potential Common Method Bias (CMB) cannot be fully ruled out. Although procedural remedies were implemented during the survey design phase such as ensuring respondent anonymity and counterbalancing item order, and Harman's single-factor test confirmed that CMB did not pose a substantial threat to our dataset (the first factor accounted for 32.5% of the total variance, which is well below the 50% threshold established for this test), future research could adopt multi-source data or objective behavioral logs to further mitigate common response biases. Third, with respect to causal inference, due to the observational and cross-sectional nature of the dataset, the empirical relationships identified by XGBoost and other predictive models reflect statistical associations and predictive importance rather than definitive causal mechanisms. Although non-parametric tree ensembles can effectively capture non-linear feature sensitivities, establishing rigorous causality requires future research using longitudinal panel tracking, difference-in-differences (DID) estimation, or quasi-experimental designs to capture the dynamic evolution of adoption behaviors over time. Future research can also conduct comparative analysis to further improve the depth and reliability of this study.

## Data Availability

The raw data supporting the conclusions of this article will be made available by the authors, without undue reservation.
