# Supplementary material for: Exploring care adoption intention among older adults using XGBoost: a rural case in Anhui, China
Source: Front Public Health. 2026 Aug 20;14:1920052. doi: 10.3389/fpubh.2026.1920052 (PMC13538432; doi:10.3389/fpubh.2026.1920052)
Supplement: Supplementary file 1 [file Table_1.docx]

**Supplementary information for**

Exploring elderly care adoption intention using XGBoost: A rural case in Anhui, China

**Appendix A**

**Sampling method**

1. Population definition

The target population of this study is rural residents aged 60 and above in Huainan City, Anhui Province. Multi-stage stratified sampling was adopted to reduce subjective selection bias and improve sample representativeness. Rural villages in Huainan were stratified by three indicators: per capita disposable income of rural residents, number of village-level elderly service stations and agricultural industrial scale, and divided into three types, as shown in Table S1.

Table S1. Regional division and statistics

| Division | Covered counties/districts | Total rural elderly population | Percentage |
| --- | --- | --- | --- |
| Region I (Suburban urban fringe villages) | Tianjia’an District, Datong District, Xiejiaji District, Bagongshan District | 128,830 | 12% |
| Region II (Agriculture and mining mixed towns) | Panji District, Maoji Experimental District, Fengtai County | 578,724 | 54% |
| Region III (Remote large agricultural county) | Shouxian County | 366,046 | 34% |

2. Three-stage sampling procedure

Stage 1: Stratification of rural areas in Huainan

According to economic and pension resource indicators, rural areas of Huainan are classified into three strata:

Suburban villages (Tian’an District adjacent to urban area);

Plain agricultural towns (Panji District, Fengtai County);

Remote rural towns (Shouxian County).

The number of villages selected from each stratum is proportional to the local elderly population.

Stage 2: Village-level proportional sampling

Within each stratum, villages were randomly selected proportional to the size of the rural elderly population. More questionnaires were allocated to villages with a larger aging population to guarantee population representativeness.

Stage 3: On-site systematic sampling

Three fixed rules were followed to avoid location and time sampling bias during face-to-face survey:

(1) With the coordination of village committee staff, we conducted door-to-door interviews with all registered elderly residents aged 60 and above in sampled villages. Village cadres accompanied investigators to each household to identify eligible older adults and explain the survey purpose.

(2) For households where the elderly were temporarily out, we supplemented questionnaires at four fixed public sites within the village, including the village committee square, rural supermarket, village entrance and senior activity room. One eligible older adult was invited to fill in the questionnaire every 5 minutes; if the interviewee refused, the nearest available elderly resident was selected as a substitute.

(3) The sampling location was rotated every two hours to avoid over-concentrated sampling and reduce location bias.

3. Sample size calculation

To calculate the minimum sample size of this rural questionnaire survey, we adopt the sample size formula based on the central limit theorem:

$n=\frac{Z^{2}\cdot p\cdot(1-p)}{E^{2}}$ (1)

$n_{adj}=\frac{n}{1+\frac{n-1}{N}}$ (2)

where $n$ is the initial calculated sample size; $n_{adj}$ is the adjusted sample size after finite population correction; $Z$ is the Z-score matching the target confidence level; $p=0.5$means the conservative estimated proportion to maximize sample size demand; $E=0.05$ means the margin of error; $N=1,073,600$ denotes the total rural population of Huainan City. The adjusted sample size under the 95% confidence level is 306. We distributed 336 questionnaires in the field survey and obtained 320 valid samples, which exceeds the theoretical minimum requirement and guarantees statistical robustness.

**Appendix B**

**Questionnaire content**

Survey introduction

Dear respondents,

This questionnaire aims to investigate rural older adults’ behavioral intention to adopt smart mutual-aid elderly care services (e.g., one-touch emergency call devices, health monitoring terminals, online neighborhood mutual-aid platforms), and identify key influencing factors. The findings will provide empirical evidence to optimize rural smart elderly care systems and improve elderly well-being.

All responses are anonymous. Your personal information will be strictly confidential and only used for academic analysis. No professional knowledge is required; please select answers based on your real experience. Thank you for taking your valuable time to participate in this survey. Wish you good health and a happy life!

Part 1 Basic demographic information

1. Gender

□ Male	□ Female

2. Age: ____ years old
3. Educational attainment

□ Illiterate	□ Primary School	□ Junior High School

□ High School / Technical Secondary School	□ Junior College and Above

4. Self-reported health status

□ Poor health requiring full-time care from others

□ Sub-health needing occasional assistance in daily life

□ Generally healthy with minor ailments and full self-care ability

□ Very healthy and fully independent in daily activities

5. Living arrangement

□ Living alone (no family members co-reside)

□ Living with spouse, children or other relatives

6. Have you ever heard of rural smart mutual-aid elderly services (one-touch emergency call, intelligent health monitoring, online neighborhood assistance, etc.)?

□ Yes	□ No

(If you select “No”, please skip to the end of the questionnaire)

Part 2 Core measurement items

Please tick the option that matches your real feelings.

5-point Likert scale:

1 = Strongly Disagree, 2 = Disagree, 3 = Neutral / Uncertain, 4 = Agree, 5 = Strongly Agree

(1) Perceived Ease of Use (PEU)

1. It is simple for me to learn and operate smart mutual-aid elderly care services such as one-touch emergency call bracelets.

□ 1	□ 2	□ 3	□ 4	□ 5

2. I can easily operate smart elderly care devices with large buttons and voice control functions.

□ 1	□ 2	□ 3	□ 4	□ 5

3. I can independently use these smart elderly services without guidance from others.

□ 1	□ 2	□ 3	□ 4	□ 5

(2) Perceived Usefulness (PU)

1. Smart mutual-aid elderly care services can solve my practical pension-related problems.

□ 1	□ 2	□ 3	□ 4	□ 5

2. Using these smart services can improve my daily convenience and personal safety.

□ 1	□ 2	□ 3	□ 4	□ 5

3. Smart elderly care services can reduce my reliance on children or other caregivers.

□ 1	□ 2	□ 3	□ 4	□ 5

(3) Social Support (SS)

1. Neighbors are willing to help me learn to use smart elderly care equipment.

□ 1	□ 2	□ 3	□ 4	□ 5

2. Village cadres can provide operational guidance and support for these smart services.

□ 1	□ 2	□ 3	□ 4	□ 5

3. My children will assist me in setting up and operating smart elderly care platforms and devices.

□ 1	□ 2	□ 3	□ 4	□ 5

(4) Behavioral Intention (BI)

1. I am willing to try smart mutual-aid elderly care devices and services.

□ 1	□ 2	□ 3	□ 4	□ 5

2. I will continuously use smart mutual-aid elderly care services in the future.

□ 1	□ 2	□ 3	□ 4	□ 5

3. I will recommend these smart elderly care services to other rural elderly residents around me.

□ 1	□ 2	□ 3	□ 4	□ 5

Part 3 Optional comments & suggestions

Do you have any other thoughts or suggestions regarding the use or promotion of rural smart mutual-aid elderly care services?

Closing remarks

Thank you sincerely for completing this questionnaire! Wish you a long and happy life with your family.
